# The urgent need to integrate infectious diseases expertise into the management of cancer patients with infections in sub-Saharan Africa in the era of antimicrobial resistance: a policy brief

**DOI:** 10.3389/fphar.2026.1889223

**Published:** 2026-08-20

**Authors:** Margaret Lubwama, Elizabeth Gulleen, Simon Sekyanzi, Benon Asiimwe, Alhaji Njai, Jody Winter, Lesley Hoyles, Nixon Niyonzima, Jackson Orem, Henry Ddungu, Joyce Kambugu, Annet Nakaganda, David P. Kateete, Warren Phipps, Freddie Bwanga

**Affiliations:** 1 Department of Medical Microbiology, School of Biomedical Sciences, College of Health Sciences, Makerere University, Kampala, Uganda; 2 Division of Infectious Diseases and Internal Medicine, University of Minnesota, Minneapolis, MN, United States; 3 Uganda Cancer Institute, Kampala, Uganda; 4 Department of Biological Sciences, Fourah Bay College and Department Microbiology, College of Medicine and Allied Health Sciences, University of Sierra Leone, Freetown, Sierra Leone; 5 Koinadugu College, Kabala, Sierra Leone; 6 Department of Biosciences, School of Science and Technology, Nottingham Trent University, Nottingham, United Kingdom; 7 Ananda Center for Cancer Research, Kampala, Uganda; 8 Department of Immunology and Molecular Biology, School of Biomedical Sciences, College of Health Sciences, Makerere University, Kampala, Uganda; 9 Vaccine and Infectious Disease Division, Fred Hutchinson Cancer Center, Seattle, WA, United States; 10 Allergy and Infectious Diseases Division, Department of Medicine, University of Washington, Seattle, WA, United States

**Keywords:** antimicrobial resistance, cancer, multidrug-resistant, national cancer control plan, sub-Saharan Africa, Uganda

## Abstract

Antimicrobial resistance (AMR) is a global health concern. Cancer patients are 1.5–2 times more likely to be affected by AMR than other patient groups. This policy brief, informed by microbiological studies conducted at the Uganda Cancer Institute since 2014, presents key recommendations and implementation strategies for the management of bacterial infections in cancer patients in sub-Saharan Africa (SSA). Our recommendations include strengthening AMR surveillance, implementing context-specific infection prevention and control and antimicrobial stewardship programs, and raising awareness of AMR in cancer in the health sector and community in SSA. A collaborative multidisciplinary approach which includes infectious diseases expertise is required to effectively implement health policies and guidelines. Proactive and effective health policies which address AMR in cancer are critical in cancer centers in SSA.

## 1 Introduction

Antimicrobial resistance (AMR) is a global health concern. In 2021, 4.71 million deaths were associated with AMR globally. By 2050, it is estimated that 1.91 million deaths will be attributable to AMR and 8.22 million deaths will be associated with AMR (GBD 2021 Antimicrobial Resistance Collaborators, 2024). Low- and middle-income countries (LMICs) are projected to be disproportionately affected by AMR (GBD 2021 Antimicrobial Resistance Collaborators, 2024; World Bank, 2017). In Uganda, there is an increase in the rates of AMR observed in the national AMR surveillance data over the years (Namubiru et al., 2024). Additionally, the number of AMR-attributable deaths in Uganda is higher than deaths from respiratory infections and tuberculosis, cardiovascular diseases, HIV/AIDS and sexually transmitted infections, neglected tropical diseases and malaria, and cancer (Institute for Health Metrics and Evaluation IHME, 2022).

AMR disproportionately affects cancer patients with a prevalence estimated to be 1.5 to 2 times higher in bacteria isolated from hospitalized patients with cancer compared to those without cancer (Gupta et al., 2024). AMR poses a significant threat to cancer care by complicating management of infections in patients with cancer (Edward and Owoicho, 2026; Nanayakkara et al., 2021). Cancer patients are prone to infections due to the immunosuppressive effects of cancer and chemotherapy (Lewis et al., 2011; Vento and Cainelli, 2003). To reduce the risk of infection due to immunosuppression, chemotherapy dosages are reduced and treatment delayed or switched to less toxic regimens which may be less effective, leading to poor outcomes including increased morbidity and mortality among cancer patients (Lalami and Klastersky, 2017). Therefore, addressing the threat of AMR in the cancer population is critical. To successfully identify, treat and prevent infections in patients with cancer, recognition of risk factors associated with and pathogens causing infections is crucial. Additionally, knowledge of the susceptibility patterns of the bacterial pathogens to antibiotics is necessary to guide antimicrobial management. Clinicians must, therefore, have up-to-date knowledge of the changing epidemiology of infections in their institutes (Rolston, 2014; Gudiol et al., 2013).

Over the years, high-income countries (HICs) have observed trends in bacterial causes of infections in their cancer populations. In the early 1960s the main causes of infection were Gram-negative bacteria. To counter this, fluoroquinolone prophylaxis was introduced to prevent infections during cancer treatment. Together with the introduction of central venous catheters, this predisposed cancer patients to infections caused by Gram-positive bacteria (Viscoli et al., 2005). More recently, multidrug-resistant (MDR) bacteria have become more common causes of infection among cancer patients, reflecting the global AMR challenge (Gudiol et al., 2013). Through multidisciplinary collaborative approaches between oncologists and infectious diseases teams, infection management algorithms and guidelines based on the epidemiology of infections have been created to meet this challenge in HICs (Taplitz et al., 2018). Infectious diseases teams including microbiologists, infectious diseases doctors, pharmacists, infection prevention specialists, and public health specialists support the development of robust infection prevention and control (IPC) and antimicrobial stewardship (AMS) programs in cancer centers.

There is a paucity of data on infections and AMR in cancer in Africa. Majority of the studies in sub-Saharan Africa (SSA) have not yet demonstrated trends in the causative organisms driving infections in their cancer populations over the years (Obadare et al., 2025). Furthermore, the burden of AMR in cancer has not been fully elucidated. Additionally, identification of patients with the highest risk of infection has been difficult due to the nature of the designs of the few studies (Obadare et al., 2025). This is compounded by the unique AMR challenges faced in SSA including limited access to microbiological diagnostics and safe antibiotics, coupled with the presence of limited infectious diseases expertise to guide the development of diagnostic and antibiotic management algorithms (Sartorius et al., 2024). Due to the limited data on causes of infections in cancer, centers in SSA use infection management guidelines that have been developed in HICs, and yet it is likely that the causes and characteristics of infections in Africa are distinct from those in HICs.

This policy brief presents key recommendations for the management of infections in cancer patients in SSA. These policy recommendations emerge from microbiological studies which determined the causes of bacteremia and their antimicrobial susceptibility profiles at the Uganda Cancer Institute (UCI). We also describe steps which cancer centers can take to implement the suggested recommendations. We need to prioritize data collection and surveillance of AMR in cancer in SSA. Inclusion of infectious diseases expertise including microbiologists, infectious diseases doctors, pharmacists, infection prevention specialists, and public health specialists as leaders in the field of AMR to collaboratively support cancer care experts in the management of infections in patients with cancer in SSA can significantly improve patient outcomes. Infectious diseases teams can develop, implement and evaluate context-specific surveillance, IPC and AMS programs within cancer centers in SSA.

## 2 Methods

We used a narrative review approach to synthesize evidence on AMR in cancer at UCI and identify priority areas for key policy recommendations for the management of infections in cancer patients in SSA. We included all observational studies on infection at UCI from 2014–2022 (Gulleen et al., 2023; Lubwama et al., 2024a; Lubwama et al., 2019; Lubwama et al., 2024b). We used a thematic analysis approach and categorized the data into the following themes: incidence and prevalence of bacteremia, organisms causing bacteria, and multi-drug resistance.

## 3 Analysis

### 3.1 Incidence and prevalence of bacteremia in patients with cancer at the Uganda cancer institute

Bacteremia studies in patients with cancer at the UCI were carried out in 2014 including pediatric and adult patients with hematologic malignancies and solid tumors, 2017-2020 including pediatric and adult patients with hematologic malignancies and 2022 including adults with solid tumors (Table 1). Bacteria were isolated from the bloodstream of a higher proportion of UCI patients with hematologic malignancies compared to patients with solid tumors. In 2022, the incidence rate of bacteremia was 12% (12/104). In the study from 2017-2020, the incidence rate of bacteremia was 33% (43/132). In 2014, the prevalence rate of bacteremia among patients with hematology malignancies was 20% (17/87) while the prevalence rate of bacteremia among patients with solid tumors was 13% (6/45).

**TABLE 1:** Bacteremia studies in patients with cancer at the Uganda Cancer Institute (UCI).

Period	Study design	Cancer type	Age	Neutropenia N, (%) (out of the total number of patients)	Incidence/Prevalence of bacteremia N, (%)	No. of bacterial isolates characterized	GN (%)	GP (%)	Ref
2022	Prospective cohort	Solid	Adults	40/104 (38)	12/104 (12)*	13	75	25	Gulleen et al. (2023)
2017-2020	Prospective cohort	Hematology	Pediatrics, adults	132/132 (100)	43/132 (33)*	64	78	22	Lubwama et al. (2024a)
2014	Cross sectional	Hematology	Pediatrics, adults	50/87 (58)	17/87 (20)**	26	77	23	Lubwama et al. (2019)
2014	Cross sectional	Solid	Pediatrics, adults	12/45 (27)	6/45 (13)**	7	29	71	Lubwama et al. (2019)

* Incidence; **Prevalence; GN: Gram-negative; GP: Gram-positive.

### 3.2 Organisms causing bacteremia at the Uganda cancer institute

Gram-negative bacteria persistently dominated as causative agents of bacteremia at UCI in the studies since 2014 (Gulleen et al., 2023; Lubwama et al., 2024a; Lubwama et al., 2019). This is expected from centers in LMIC settings which do not have predisposing factors for Gram-positive bacteremia such as use of central venous catheters. The most frequently isolated Gram-negative bacteria from cancer patients with bacteriaemia (n = 82) were Enterobacterales (n = 77, 94%), most commonly *Escherichia coli* (n = 42, 55%) and *Klebsiella pneumoniae* (n = 27, 35%). Among the Gram-positive bacteria, *Enterococcus* spp. were the most common (n = 9/28, 32%).

### 3.3 Multidrug-resistant bacteria isolated at the Uganda cancer institute

**FIGURE 1:**
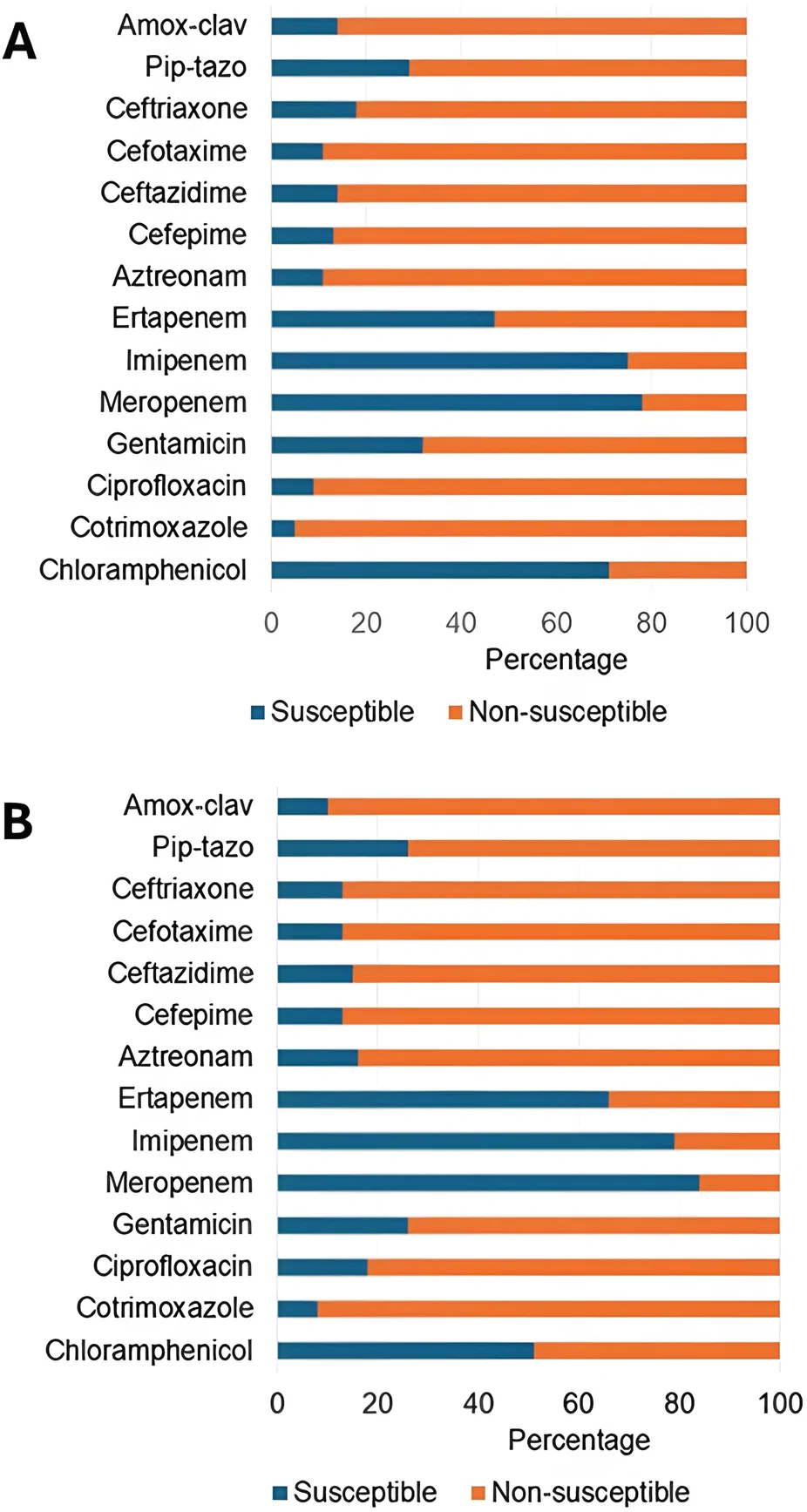
Proportion of *E. coli* **(A)** (n = 62) and *K. pneumoniae* **(B)** (n = 43) isolates that were susceptible and non-susceptible to various antibiotics at the UCI. Amox-clav: amoxicillin-clavulanic acid; Pip-tazo: piperacillin-tazobactam.

Figure 1 shows the antimicrobial susceptibility patterns of all *E. coli* and *K. pneumoniae* isolates from cancer patients with bacteriaemia at the UCI in 2014, 2017-2021, 2022.

Over 80% of the bacteria causing bacteremia at the UCI were MDR which was defined as an isolate being non-susceptible to at least one agent in ≥3 antimicrobial categories. Fewer than 20% of the *E. coli* and *K. pneumoniae* isolates were susceptible to the third-generation cephalosporins. More than 70% of Enterobacterales had the extended spectrum beta-lactamase (ESBL) phenotype. Genotyping of Enterobacterales isolated between 2017 and 2021 showed CTX-M to be the most predominant ESBL (Lubwama et al., 2024b). The antibiotic of choice for ESBL-producing Enterobacterales is a carbapenem. However, our studies have shown that over 20% of Enterobacterales isolates at the UCI were resistant to at least one carbapenem. Chloramphenicol, which shows almost comparable susceptibility results with carbapenems compared to the other antibiotics, is one of the antibiotics recommended for the treatment of septicemia in adults in the Uganda Clinical Guidelines UCG (Ministry of Health MOH U, 2024). However, careful consideration is needed for its use in patients with hematologic malignancies since it can cause bone-marrow suppression as a side effect (West et al., 1988). The other recommended treatment for septicemia in the UCG is gentamicin and cloxacillin. However, there is low susceptibility of our isolates to both aminoglycosides and the penicillins (less than 50% susceptible).

## 4 Policy implications

Our studies on bacteremia at the UCI are consistent with the global AMR challenge that has been observed elsewhere. Given the growing burden that AMR places on cancer, proactive management of AMR is urgently needed if cancer outcomes are to improve. Therefore, effective health policies which address AMR in cancer are critical in cancer centers in SSA.

The studies on bacteremia in patients with cancer at the UCI were carried out in 2014, 2017-2021, and 2022, with no data in between these years (Gulleen et al., 2023; Lubwama et al., 2024a; Lubwama et al., 2019). Gaps in data are a common phenomenon in LMICs (Iskandar et al., 2021). Data which depend on microbiology diagnostics, including molecular diagnostics, rely on funded research studies. Additionally, diagnostic microbiology is not usually available to patients in LMICs due to limited access to microbiology laboratories and the costly nature of the tests (Sorn, 2026; Gandra et al., 2020; Gulleen and Lubwama, 2024). Consequently, lack of data limits the effective surveillance of infections in cancer (Iskandar et al., 2021). More consistent use of diagnostic microbiology is needed to guide the care of cancer patients with infections in SSA, and to generate comprehensive and ongoing surveillance datasets.

The high rates of MDR bacteria observed in the studies at UCI have significant implications for IPC and AMS programs. MDR bacteria have also been observed in other cancer centers in SSA (Worku et al., 2022; Zain et al., 2022; Chinowaita et al., 2020; Mvalo et al., 2018). Center-specific updated guidelines for infection management of cancer patients in SSA which considers the epidemiology of organisms at the center are urgently needed. Additionally, training healthcare workers in those guidelines is crucial. It is important to note that the presence of MDR bacteria may require the use of effective and quality high-end antibiotics which may be inaccessible to cancer centers in LMICs (Sartorius et al., 2024).

To be effectively utilized, infectious diseases data require interpretation by infectious diseases experts. Through a multidisciplinary approach, infectious diseases and cancer care experts should collaborate to ensure comprehensive management of infections in patients with cancer in SSA (Taplitz et al., 2018). Our studies were carried out at the Makerere University Clinical Microbiology Laboratory and were led by microbiology and infectious diseases teams working collaboratively with UCI oncologists. UCI lacked blood culture microbiology facilities and infectious diseases expertise at that time. This gap may reflect the observations in other cancer centers in SSA and demonstrates the need for proactive collaboration. The infectious diseases team should take the lead in developing strategies in cancer centers in SSA which are aligned with the World Health Organization global action plan on AMR and country AMR national action plans (World Health Organization, 2026).

### 4.1 Key recommendations derived from our UCI studies

- Strengthen AMR surveillance systems, including genomic surveillance, to fill knowledge gaps on causative organisms, antimicrobial use, resistance mechanisms, rare and emerging pathogens, and transmission patterns of MDR bacteria.
- Raise awareness of AMR in cancer in the healthcare sector and the community.

#### 4.1.1 Actions to ensure the above recommendations are met include

- Establishing links and permanent positions for infectious diseases experts including microbiologists, infectious diseases doctors, pharmacists, and public health specialists in cancer centers in SSA to lead and drive infectious diseases and AMR policies to ensure continuous monitoring of AMR trends over time.
- Developing strategies, guidelines and checklists for AMR surveillance.
- Obtaining financial commitment from cancer centers in SSA to ensure continuous AMR surveillance to generate local antibiograms and show trends over time.

### 4.2 Other key recommendations and future policy proposals

- Implement context-specific robust IPC programs to prevent the spread of MDR bacteria.
- Develop AMS programs with antibiograms to be used to design prescribing guidelines that consider the high prevalence of resistance to some of the currently recommended antibiotics.
- Develop data-informed diagnostic and antimicrobial algorithms and pathways for antimicrobial prescribing clinical guidelines in AMS programs.
- Develop electronic databases which merge clinical, microbiological/laboratory and pharmacy data. By easing and standardizing data collection, analyses and providing options for data sharing, electronic databases will support the monitoring and evaluation of AMR surveillance, IPC and AMS programs developed in cancer centers in SSA.
- Commit to research focused on AMR in cancer, such as evaluation of rapid diagnostics to inform diagnostic stewardship and AMS policies and guidelines.
- Develop curricula and short courses on AMR for both the cancer care team and the community, and incorporating courses on management of infection in cancer patients into the education of clinical microbiologists, pharmacists, and public health specialists.

While the recommendations suggested are aligned with the WHO global action plan on AMR objectives, it is important to note that there may be implementation barriers in many SSA settings. Some of the challenges that may be faced in SSA include weak healthcare systems and limited access to resources such as microbiological diagnostics, safe antibiotics, and infectious diseases expertise to guide the development of diagnostic and antibiotic management algorithms (Sartorius et al., 2024). The above recommendations will require national financial commitment to invest in the study and effective management of infectious diseases in cancer centers in SSA.

### 4.3 Incorporating AMR into national cancer control plans in SSA: a framework

Given the high burden of MDR infections in patients with cancer, the WHO and the Union for International Cancer Control (UICC) recommends AMR to be incorporated into country national cancer control plans (NCCP) for better cancer control (UICC, 2026). NCCPs are country-specific frameworks and documents to address cancer care and can provide strategic guidance to tackle country-specific AMR burdens in countries in SSA. Therefore, incorporating AMR into NCCPs will tailor recommendations to the local context, given the regional heterogeneity in infectious diseases capacity including laboratory human and structural resources, and access to antibiotics (Moyo et al., 2023). According to the UICC only 2 out of 98 countries have included consideration of antibiotic options for treatment of infections in their NCCPs (UICC, 2026). Turkey highlights the use of antibiotics for managing *H. pylori*, a bacterium associated with gastric cancers. Zambia identifies lack of guidelines in the monitoring of antibiotic use and realizes the WHO AMR and antibiotic use guidelines as an opportunity for guidance on antibiotic use and surveillance (T.R. Ministry of Health, 2026; Ministry of Health, 2026). However, there remains gaps in AMR-specific considerations to address the AMR burden in cancer.

**FIGURE 2:**
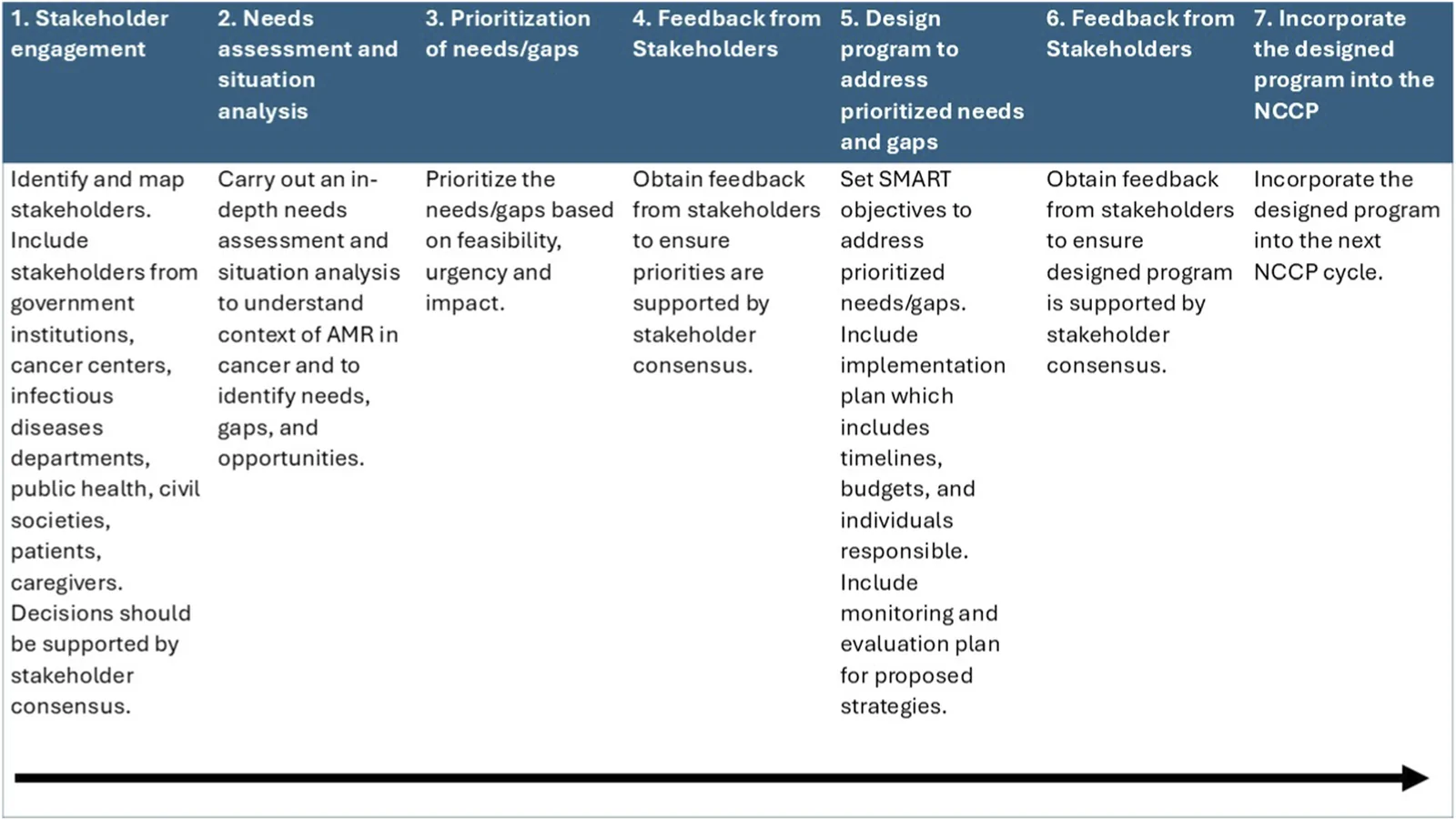
A framework to incorporate AMR into national cancer control plans in countries in SSA.

The process of how to incorporate AMR into NCCPs is seldom addressed. We present a framework to incorporate AMR into NCCPs in countries in SSA (Figure 2). Incorporating AMR into NCCPs in SSA will enable cancer care providers to systematically and practically address the growing burden of AMR in cancer. For the success of such a project a multidisciplinary team responsible for collaborative decision-making will ensure that outcomes are geared towards comprehensive quality of care.

#### 4.3.1 Stakeholder engagement

Stakeholder engagement is critical to address AMR in cancer in SSA. Key stakeholders should be included and participate in all steps of the process of incorporating infection management in AMR into NCCPs. Decisions should be supported by stakeholder consensus. Stakeholders should include at least representatives from government institutions and ministries, cancer centers, infectious diseases and microbiology departments, public health, civil societies and community (including patients and their caregivers).

#### 4.3.2 Needs assessment and situation analysis

An in-depth needs assessment and situation analysis enables us to understand the context of infection and AMR in cancer in a country. It systematically identifies the burden of infection and AMR in cancer, needs, gaps, and factors and causes associated with the gaps in the current infection management practices in cancer centers in a country. This will in turn guide decision-making, resource allocation and strategy planning by stakeholders to address the challenges and achieve what is desired. Information on infection and AMR in cancer in countries can be obtained from data from cancer centers and publications. The WHO Global Action Plan on AMR and country national action plans on AMR are valuable tools which can be used to determine the desired outcome for infection management and AMR in cancer. A coordinated joint needs assessment including stakeholders from most of the cancer centers in a country should be undertaken to ensure that majority of the country’s experiences on infection and AMR in cancer are captured to inform the NCCP.

#### 4.3.3 Prioritization of needs/gaps

Prioritization of needs is a critical step prior to designing a program to address the infection management and AMR needs/gaps identified in the needs assessment. Prioritization should be based on urgency, feasibility and impact. For example, the needs/gaps identified in the needs assessment can include:

- Gap 1: observations of high rates of infections but no surveillance data.
- Gap 2: cancer centers unaware of which antibiotics to use.
- Gap 3: no IPC measures in place.
- Gap 4: lack of human resources, e.g., infectious diseases specialists including laboratory scientists, microbiologists, doctors, pharmacists and public health specialists.
- Gap 5: lack of infrastructure, e.g., laboratories.
- Gap 6: lack of awareness and knowledge of infection and AMR among health workers, patients and caregivers.

When prioritizing these needs, Gap 5 and Gap 4 may be higher in priority than Gaps 1, 2, 3 and 6 as a starting point for cancer centers in SSA. Infrastructure is needed to generate data which will feed into AMR surveillance, AMS, IPC, and educational programs.

#### 4.3.4 Designing a program to address the needs

**FIGURE 3:**
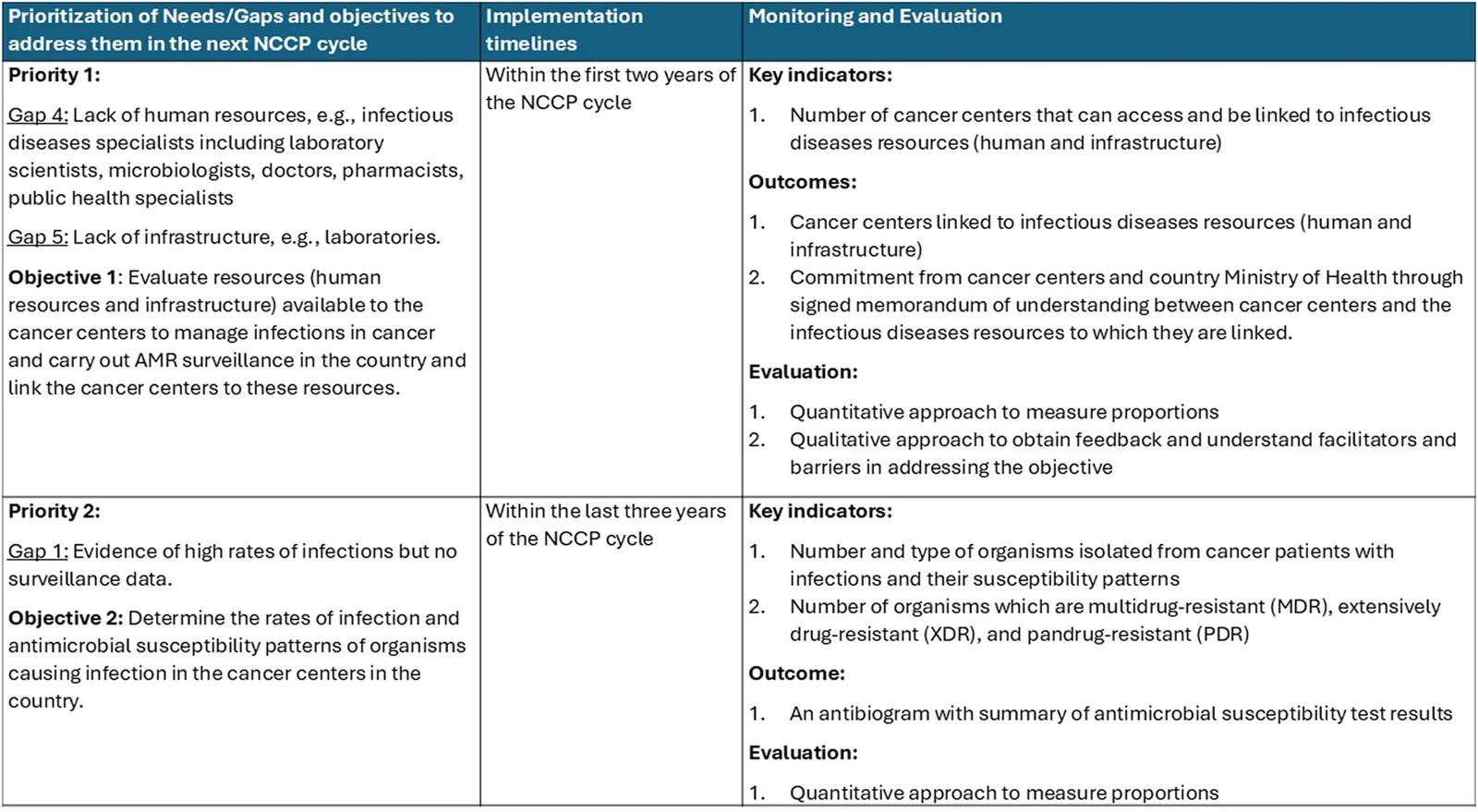
An example of prioritization of needs and setting objectives to address the needs/gaps. Priority 1 addresses the resources (human and infrastructure) available to cancer centers to manage infections and carry out AMR surveillance. Priority 2 addresses AMR surveillance. Objectives 1 and 2 address Priorities 1 and 2 in the next 5-year NCCP cycle.

The objectives set for the program should, therefore, be specific, measurable, achievable, relevant, and time-bound (SMART), and directly related to the prioritized gaps. It is important to define objectives with the relevant key stakeholders whose experience is important for context. In the example above, a country may choose to set objectives which address Gaps 4, 5 (Priority 1) and Gap 1(Priority 2) in the next NCCP cycle (Figure 3). Additionally, the designed program should have an implementation plan which includes the timelines, budgeting and the individuals responsible. Importantly, the program designed should have a monitoring and evaluation plan to determine if the proposed strategies are being implemented and outcomes achieved. Feedback from relevant key stakeholders is critical before incorporating the designed program into the NCCP. Overall, the infection management and AMR program should be cost-effective and affordable if it is likely to be implemented.

## 5 Limitations

This policy brief is limited by evidence derived from observational studies from a single cancer center (UCI). However, it addresses the global pertinent and emerging concern of AMR in cancer, highlighting the critical need for infectious diseases expertise in cancer centers in SSA, and provides a practical framework for incorporating AMR into NCCPs. Given the scarcity of data on infections and AMR in cancer centers across SSA, using the practical framework we have shared, the different countries in SSA should derive recommendations for their cancer centers from their own experiences.

## 6 Conclusion

AMR in cancer is a challenge even in cancer institutes in SSA. Given the growing burden that AMR places on cancer, management of AMR is key if cancer outcomes are to improve. To effectively implement health policies which address AMR in cancer centers in SSA, a collaborative multidisciplinary approach including infectious diseases expertise is paramount. Proactive and effective health policies which address AMR in cancer are critical in cancer centers in SSA.
